# Differences in Daytime Activity Levels and Daytime Sleep Between Night and Day Duty: An Observational Study in Italian Orthopedic Nurses

**DOI:** 10.3389/fphys.2021.628231

**Published:** 2021-02-26

**Authors:** Eliana Roveda, Lucia Castelli, Letizia Galasso, Antonino Mulè, Emiliano Cè, Vincenzo Condemi, Giuseppe Banfi, Angela Montaruli, Fabio Esposito

**Affiliations:** ^1^Department of Biomedical Sciences for Health, University of Milan, Milan, Italy; ^2^IRCCS Istituto Ortopedico Galeazzi, Milan, Italy

**Keywords:** daytime activity levels, actigraphic monitoring, daytime sleep, occupational medicine, shift work nurses, sleep, chronotype

## Abstract

Working nonstandard work schedules is often associated with increased sedentary behavior and risk of sleep disorders. Night shift workers are prone to accumulating sleep debt, which they recover by sleeping during the day. The effect on daytime activity levels is unknown. The present study aims to objectively assess whether daytime sleep could affect daytime activity levels of shift worker nurses, resulting in an accumulation of their activity debt differently between working and rest periods. The study population (*N* = 37; mean age 41.7 ± 9.1 years) was composed of orthopedic nurses working on a rotating schedule, including either a night shift (NS) or only day/afternoon shift (DS). Actigraph monitoring lasted both on the working and the rest period. For the NS nurses, the working period recorded higher daytime activity levels than the rest period, while daytime sleep during the working and rest periods was similar. Conversely, DS nurses showed higher daytime activity levels and shorter daytime sleep during the working period. NS nurses were less active than DS nurses during the working period, probably because NS tended to have a longer daytime sleep. During the rest period, daytime activity levels for both groups were decreased. For NS nurses, sleep recorded the better sleep parameters during the rest period, while sleep parameters did not show significant differences between the working and the rest periods in DS. During the working period, NS nurses slept worse than the DS nurses. Both groups tended to accumulate a debt in daytime activity levels during the rest period. While daytime sleep may be an excellent way to counteract sleep debt and increase sleep duration over 24 h period, on the other hand, it makes nurses less active.

## Introduction

In modern society, the transport, industry, commerce, health care, and hospitality sectors require workers all day long, making the work shifts covering a period of 24 h. Shift work is commonly defined as any kind of work outside the standard working time (8:00–18:00) and includes various working time arrangements (Costa, 2003). According to recent estimates, 21% of the European Union workforce is employed in a nonstandard work schedule (Parent-Thirion et al., 2017). Since hospitals operate around the clock, nursing staff works continuous working schedules (Li et al., 2019).

Studies on shift work have largely investigated exposure to health risk factors. Working irregular hours and night shifts increases the risk of hypertension (Rotenberg et al., 2016), cardiovascular events, type 2 diabetes (Kecklund and Axelsson, 2016; Strohmaier et al., 2018), obesity, metabolic syndrome (Wang et al., 2014), melatonin and cortisol imbalance (Bhatti et al., 2014; Niu et al., 2015; Strohmaier et al., 2018), and cancer (Kecklund and Axelsson, 2016; Aneja, 2019). The main mechanism implicated in these disorders is the disruption of circadian rhythm (Roveda et al., 2019; Galasso et al., 2020) because the atypical working times are not aligned with a worker’s endogenous clock (Hittle and Gillespie, 2019).

Health is not the only dimension negatively affected by rotating through shifts. The continuous alternation in the work schedule can interfere with daily activity levels. Some studies have shown that greater physical activity can improve biological rhythm parameters, sleep quality, general and neuromuscular fatigue, work-related stress, and adaptation to changes in shift schedule (Atkinson et al., 2008; Cè et al., 2020). The benefits of physical activity on physical and mental health are manifold (Warburton, 2006), as are the harmful effects of inactivity (Blair et al., 2012). Despite awareness about the importance of maintaining an active lifestyle, shift workers encounter many organizational and motivational barriers to engaging in an adequate daily activity level (Atkinson et al., 2008; Alves et al., 2017). Night shift workers are noted to have a more sedentary lifestyle and less opportunity for regular physical activity than do day shift workers (Neil-Sztramko et al., 2016; McElroy et al., 2020). In studies on occupational health and daily activity, Peplonska et al. (2014) and Neil-Sztramko et al. (2016) reported higher or similar daily activity levels for night and day shift workers, but this could have been the result of occupational, physical activity (Peplonska et al., 2014; Neil-Sztramko et al., 2016).

To date, physical activity assessment has been based on data gleaned from self-report questionnaires and rarely through objective assessment *via* actigraphic monitoring of daily activity (Marqueze et al., 2014; Peplonska et al., 2014; Alves et al., 2017). This non-invasive method of monitoring human rest/activity cycles can provide a more accurate measurement of daily activity levels. In their recent study, Chang and Li (2019) used actigraphy to measure daily activity levels in a cohort of Taiwanese nurses working different shifts (day, evening, and night). No differences in daytime activity levels among the three work shifts were found; however, lower activity levels during workdays were associated with poor sleep quality, as measured by the Pittsburgh Sleep Quality Index questionnaire (Chang and Li, 2019).

Sleep is another lifestyle sphere usually affected by rotating and night work. Shift work and disruption of circadian rhythm perturb the regular sleep-wake cycle, reducing sleep quality in night shift workers (Åkerstedt and Wright, 2009). In most previous studies, night shift workers, including nurses, reported low sleep quality, shorter sleep duration, more significant sleep debt accumulation, and a greater need for sleep recovery compared to regular day shift workers (Park et al., 2000; Åkerstedt and Wright, 2009; Niu et al., 2015; Kecklund and Axelsson, 2016; van de Ven et al., 2016; Chang and Li, 2019). Sleep loss may lead to sleepiness during work and off-duty hours and lower vigilance and attention (Zion et al., 2018; Vanttola et al., 2019). After working the night shift, workers usually recover sleep debt with morning sleep or naps during the preceding and the consecutive afternoon (Akerstedt, 2003; Karhula et al., 2013; Korsiak et al., 2018). Another solution is workplace napping during the night shift whenever possible. Studies have used various metrics to show the benefits of napping (Ruggiero and Redeker, 2014). Some have focused on spontaneous napping (de Castro Palermo et al., 2015; Silva-Costa et al., 2017), while others have compared nap settings (Centofanti et al., 2016; Davy and Göbel, 2018). Though the merits of napping remain controversial (e.g., sleep inertia after a nap), Ruggiero and Redeker (2014) and Li et al. (2019) have recently reported on the beneficial effects of napping among nurses (Li et al., 2019). Differences in nap outcomes may result from the assessment methods: most studies have relied on self-report questionnaires and subjective assessment of sleep behavior and alertness after a nap (de Castro Palermo et al., 2015; Rotenberg et al., 2016; Davy and Göbel, 2018; Li et al., 2019). Objective data collection is seldom employed for nap analysis though recommended as a useful adjuvant to evaluate the sleep-wake cycle (Sack et al., 2007; Baek et al., 2020).

Moreover, physical inactivity and sleep debt in shift workers can be influenced by an individual’s chronotype. Chronotype refers to the phenotypic expression of internal biological rhythm, and three chronotypes are distinguished. The three chronotypes differ in several behavioral and biological aspects: morning-types are more active during the first part of the day; evening-types perform better during the second half of the day; and neither-types share features of the previous two (Montaruli et al., 2019). The more closely chronotype preferences are aligned with an individual’s work schedule, the less the negative impact of shift work on sleep quality (Hittle and Gillespie, 2019; Uekata et al., 2019). So it is not surprising that night shift work is more common among evening-types (Alves et al., 2017; McElroy et al., 2020).

With this observational study, we used actigraphy to: (i) measure daytime activity levels, daytime sleep, and sleep quality in hospital staff nurses during a working schedule divided into a working period and a rest period; (ii) determine whether the nurses regularly working the night shift modified their daytime activity levels, daytime sleep, and sleep quality between working and rest periods; (iii) investigate possible differences between night and day shift workers; and (iv) investigate potential differences between chronotypes.

## Materials and Methods

### Study Design, Participants, and Settings

The sample size and statistical power were calculated given the main objective of the study. The calculation was performed considering as a reference model, the two- or three-way ANOVA with 37 participants, the statistical power of 0.80, a value of *p* < 0.05, and an effect size of 0.45.

This study is an observational and descriptive study. The study population was composed of hospital staff nurses employed with the Galeazzi Orthopedic Institute of Milan (Italy). Recruitment took place between November 2018 and February 2019. Inclusion criteria for eligible volunteers were:

- At least 1-year regular shift work (day/afternoon or night).
- Absence of cardiovascular, endocrine, neuromuscular diseases, and pregnancy (self-reported).
- Absence of medications potentially interfering with sleep quality.
- Willingness to wear an actigraph for 5 consecutive days and to maintain a daily sleep diary.

**Figure 1:**
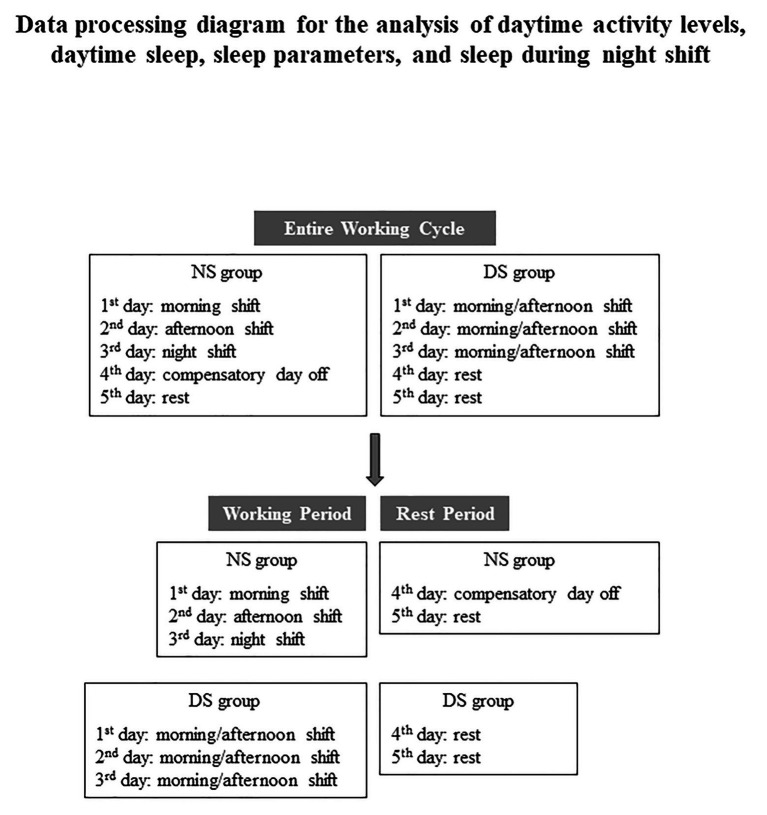
Work shift schedules for the night shift nurses (NS) and the day shift nurses (DS) grouped by the entire working cycle and working and rest periods.

The nurses worked in a two-shift system (Figure 1):

- night shift (NS): 5 continuous days in clockwise rotation: day 1 morning shift (07:00–14:00); day 2 afternoon shift (14:00–21:00); day 3 night shift (21:00–07:00); day 4 compensatory day off after night shift (day off); and day 5 rest.
- day shift (DS): 5 consecutive days [3 consecutive days, with morning/afternoon shift alternation (7:00–14:00 or 14:00–21:00)]; 2 consecutive days rest. Nurses alternate morning and afternoon shifts; they do not work the same shift for 2 consecutive days and do not work the night shift.

The study aim and procedure were explained to eligible participants: 53 gave their written, informed consent and were progressively enrolled before starting the morning shift. They completed a brief survey investigating demographic and anthropometric characteristics, health status, current medications, and responded to the Italian version of the Morningness-Eveningness Questionnaire (MEQ). To ensure accurate actigraphic monitoring, the nurses were instructed about how the actigraph unit worked and the correct compilation of the daily sleep diary. After completing one entire work shift cycle, they returned the actigraph and the diary before starting the next work cycle.

To avoid bias in the actigraphic analysis, we recruited nurses with at least one 1 year or regular shift work. We asked the nurses to maintain a regular work cycle during the actigraph monitoring avoiding shift changes.

The study was conducted under the last Helsinki Declaration’s ethical statements, approved by the Ethical Committee of the San Raffaele Hospital (CE: 156/int./2017), and registered at ClinicalTrials.gov (registration number: NCT03453398).

### Measurement

Participants filled in a form with demographic, anthropometric, and health status variables, the MEQ, and wore the actigraph for 5 days to assess daytime activity levels, nocturnal sleep parameters, daytime sleep, and sleep during night shift for the NS nurses.

#### Actigraphy

The nurses wore the Actigraphy Motion Watch 8 (CamNtech, Cambridge, United Kingdom) on the non-dominant hand’s wrist for the entire 5-day period and were allowed to remove it only when bathing or showering. Besides, during the monitoring period, each nurse recorded daily diary entries for the shift worked, bedtime, wake-up time, daytime sleep (time spent sleeping during the day), sleep during night shift for the NS nurses, and periods not wearing the actigraph. Data for the periods in which the actigraph was not worn were not included in the subsequent analysis. The data recorded by the actigraph were analyzed with Motion Ware software 1.2.28 (CamNtech, Cambridge, United Kingdom).

##### Daytime Activity Levels Assessment

A Motion Ware software program quantifies daytime activity levels. The program parameters are set to a specific period for which the daytime average activity expressed in activity counts (a.c.) is calculated. We put each day of actigraphic monitoring separately and assessed the daytime activity levels during the day, including the hours on duty and off duty.

##### Daytime Sleep

The time spent sleeping during the day (daytime sleep) was assessed through the nap analysis function (contained in the Motion Ware software) that can analyze naps or micro-sleep periods recorded by the Actigraphy Motion Watch. This function returns two main parameters: nap activity and nap duration. The first parameter identifies periods with activity counts below a set threshold, i.e., napping. The threshold was set to ≤40 activity counts (Samson et al., 2016). The second parameter identifies the nap duration, and it was set between 15 and 150 min (Samson et al., 2016). Through these two parameters, and by selecting a specific period in the actogram, the nap analysis can sense and record nap periods. We evaluated daytime sleep during off-duty and identified the periods spent to compensate for the sleep-debt resulting from the night-shift by matching the actigraphic nap periods with those entered in the daily diary.

##### Sleep Parameters Assessment

For the sleep analysis, bed- and wake-up times (data extracted from the daily diary) were entered manually into the software program, which returns values that describe the quantity and the quality of nocturnal sleep. The sleep parameters were:

- Assumed sleep: the amount of time, expressed in hours and minutes, between the beginning and the end of sleep.
- Actual sleep time: the amount of time, expressed in hours and minutes, between sleep start and sleep end. It is determined by adding up the number of epochs below the sensitivity threshold and then multiplying that value by the epoch length in minutes.
- Sleep efficiency: percentage of time in bed actually spent sleeping.
- Sleep latency: the amount of time, expressed in minutes, between sleep onset and retiring to bed. An algorithm, based on lack of movement after bedtime, automatically calculates this.
- Immobile minutes: total time, expressed in number of minutes, of no movement recorded between sleep start and end.
- Fragmentation index: the sum of the “Mobile time” (% time spent moving) and the “Immobile bouts” (% of immobile <1-min periods).

#### Morningness-Eveningness Questionnaire

Determination of chronotype was based on responses to the validated Italian version of the MEQ (Zani et al., 1984), a self-report questionnaire initially designed by Horne and Ostberg (1976). It includes 19 multiple-choice items that investigate preferences for sleep and activity, the mood before retirement or after awakening, and the time of day in which the respondent feels most active. The MEQ is extensively used as it has proven good stability (88–89) and reliability (coefficient range, 78–86); it has been translated and validated in several languages. Each response is scored between 0 and 6, and the total score ranges from 16 to 86. Three chronotypes are distinguished: evening- (E-type), neither- (N-type), and morning-type (M-type). Lower scores (range, 16–41) define the E-type dimension, differentiated in definite E-type (range, 16–30) and moderate E-type (range, 31–41); higher scores (range, 59–86) define M-type dimension, differentiated in definite M-type (range, 70–86) and moderate M-type (range, 59–69). Scores between 42 and 58 define N-types that have no particular morning or evening preferences.

### Data Processing

For each day, daytime activity levels, daytime sleep, and nocturnal sleep parameters were calculated, and the mean was calculated over several periods (Figure 1):

- Entire working cycle: all 5 days of working time.
- Working period: day 1 (morning shift 07:00–14:00), day 2 (afternoon shift 14:00–21:00), day 3 (night shift 21:00–07:00), and the 3 consecutive workdays (morning shift 07:00–14:00 or afternoon shift 14:00–21:00) for the DS group.
- Rest period: day 4 (compensatory day) and day 5 (rest) for the NS group and the 2 consecutive rest days for the DS group.

In this way, we assessed the sleep quality during the entire working cycle and the amount of time spent sleeping during the day to compensate for the low sleep quality encountered during the night shift.

By splitting the working schedule into a working and rest period, we observed whether the NS group tended to have a longer daytime sleep and, as a consequence, a reduction in the daily activity level. The differences in daytime activity levels and daytime sleep could reflect the sleep debt accumulation due to night duty or different sleep behaviors between NS and DS groups. For all working periods, daytime activity levels, daytime sleep, and sleep parameters were assessed based on the two-shift work schedule as a whole, NS and DS group, and then stratified by chronotype.

### Statistical Analysis

Normal distribution and homogeneity of variance for all response variables were tested using the Shapiro-Wilk and the Levene test. The assumptions for normality and homoscedasticity were confirmed and, based on these results, we then applied the parametric.

*T*-test investigated differences between two continuous variables [age, body mass, body-mass index (BMI), MEQ, and night naps], while the chi-square test (*χ*^2^) explored differences between categorical variables. When appropriate, two- or three-way ANOVA was applied to the three variables (daytime activity levels, daytime sleep, and sleep quality). Two-way ANOVA was used to investigate the effect of *shift typology* (NS vs. DS, between-group factor) and *chronotype* (M-type vs. N-type, within-group factor) for the entire working cycle and the working and the rest period. Three-way ANOVA was applied to check the differences for NS vs. DS (*shift typology*) and M-type vs. N-type (*chronotype*; within-group factors) during the working and the rest period (*periods*, between-group factor). The Bonferroni *post hoc* test was applied to determine statistically significant interactions.

Associations between continuous variables were evaluated using the correlation analysis with the Pearson test.

We calculated the effect size of each variable with Cohen’s effect size (*d*). A value of 0.1 indicates a *small* effect, 0.5 a *medium* effect, 0.8 a *large* effect, and > 1 a *very large* effect (Sawilowsky, 2009; Cohen, 2013).

Nurses with actigraphic or diary missing data were excluded from the analysis. No missing data were found in the demographic and MEQ questionnaires.

Statistical significance was set at <0.05. Statistical analysis was carried out using three different software applications: G Power (version 3.1.9.4, HHU- Düsseldorf, Germany) for analysis of statistical power, R Statistics (version 3.6.0, R Development Core Team, 2011), and SPSS *Statistical* Package for Social Science (version 26, IBM Corp. Released 2019. IBM SPSS Statistics for Windows, Armonk, NY: IBM Corp) for intermediate and advanced analysis.

## Results

### Anthropometric and Questionnaire Data

The study sample was 37 staff nurses (mean age, 41.7 ± 9.1 years): 24 (five men; 19 women) made up the NS group; 13 (one man; 12 women) made up the DS group. From the first group of 53 nurses, nine failed to complete actigraphy monitoring; six voluntarily withdrew from the study, and one was excluded because she was the only E-type recruited.

Table 1 presents the descriptive data for the whole sample and for each group. BMI (weight in kilograms divided by height in meters squared) was slightly above the normal range (18.50–24.99 kg/m^2^). It remained when the sample was stratified by work shift: over half of the total sample was classified as either overweight (40.5%) or obese (13.5%). The BMI of the NS group was similar to that of the total sample; 53.8% of the DS group were normal weight; the percentage of obese individuals was higher than in the NS group. The MEQ score identified a higher rate of N-types in the total sample.

**Table 1:** Anthropometric characteristics, body-mass index (BMI), and Morningness-Eveningness Questionnaire (MEQ; chronotype) for the total sample and the two groups: night shift nurses (NS) and day shift nurses (DS).

Anthropometric characteristic	Total (*n* = 37)	NS group (*n* = 24)	DS group (*n* = 13)	*p*
*Age (years)*	41.7 ± 9.1	41.1 ± 8.4	42.7 ± 10.5	n.s.
*Body mass (kg)*	70.5 ± 13.1	70.9 ± 12.1	69.7 ± 15.4	n.s.
*BMI (kg/m^2^)*	25.4 ± 4	25.2 ± 3.4	25.8 ± 5	n.s.
*Normal weight (%)*	45.9	41.7	53.8	n.s.
*Overweight (%)*	40.5	54.2	15.4	
*Obese (%)*	13.5	4.4	30.8	
**Chronotype**	**Total** **(*n* = 37)**	**NS** **(*n* = 24)**	**DS** **(*n* = 13)**	***p***
*MEQ score*	56.8 ± 7.4	57 ± 7.2	56.5 ± 8.1	n.s.
*M-types (%)*	40.5	37.5	46.2	n.s.
*N-types (%)*	59.5	62.5	53.8	

*T*-test was applied to continuous variables; the *χ*^2^ test was applied to categorical variables. n.s, not significant.

### Daytime Activity Levels and Sleep Parameters During the Entire Working Cycle

Tables 2, 3 present the data for the Entire Working Cycle analysis.

**Table 2:** Actigraphy data (mean ± SD) recorded during the Entire Working Cycle.

ENTIRE WORKING CYCLE						
	NS group			DS group		
Sleep parameters	Total (*n* = 24)	M-types (*n* = 9)	N-types (*n* = 15)	Total (*n* = 13)	M-types (*n* = 6)	N-types (*n* = 7)
Daytime activity levels (a.c.)	138.49 ± 30.57	142 ± 39.84	136.38 ± 24.81	146.7 ± 31.67	154.04 ± 35.5	139.35 ± 28.13
Daytime sleep (hours)	9.33 ± 2.96^*^	10.6 ± 3.40	8.9 ± 2.71	5.2 ± 2.58^*^	3.91 ± 2.45	5.95 ± 2.46
Assumed sleep (hours)	6.75 ± 0.8^*^	6.63 ± 0.78^**^	6.83 ± 0.8^**^	7.36 ± 1.05^*^	6.8 ± 0.61^**^	7.85 ± 1.15^**^
Actual sleep time (hours)	5.81 ± 0.62	5.71 ± 0.7	5.88 ± 0.58	6.28 ± 0.8	5.98 ± 0.41	6.55 ± 0.96
Sleep efficiency (%)	74.43 ± 6.53^*^	73.72 ± 7.64	74.86 ± 6.02	82.2 ± 8.25^*^	85.4 ± 3.48	79 ± 10.14
Sleep latency (hours)	0.68 ± 0.38^*^	0.7 ± 0.55	0.67 ± 0.28	0.26 ± 0.31^*^	0.15 ± 0.11	0.6 ± 0.41
Immobile minutes (n°)	368.69 ± 40.84^*^	361.71 ± 45.99	372.89 ± 38.49	399.96 ± 49.6^*^	380.3 ± 29.74	419.6 ± 57.92
Fragmentation Index	30.22 ± 8.03	30.1 ± 6.62^a^	30.29 ± 8.99	25.11 ± 13.13	18 ± 7.38^a^^,^ ^b^	32.3 ± 13.76^b^

Daytime activity levels measured in activity counts (a.c.); sleep parameters are reported in decimal format. Daytime sleep is calculated over the whole period (5 days); the other parameters refer to the single day. Results are presented separately for nurses working the night shift (NS) and those working only during the day (DS). The two groups are stratified by chronotype: morning-types (M-types) and neither-types (N-types).

* Two-way ANOVA – Shift typology effect.

** Two-way ANOVA – Chronotype effect.

a significant *post hoc* test for Two-way ANOVA – Shift typology × Chronotype interaction.

b significant post hoc test for Two-way ANOVA – Shift typology × Chronotype interaction.

**Table 3:** Two-way ANOVA analysis results with the two main effects (Shift typology and Chronotype) and the interaction (Shift typology × Chronotype) for the Entire Working Cycle.

ENTIRE WORKING CYCLE – Two-way ANOVA									
Variable	Shift typology effect			Chronotype effect			Shift typology × Chronotype Interaction		
	*F*	*p*	*η_p_^2^*	*F*	*p*	*η_p_^2^*	*F*	*p*	*η_p_^2^*
Daytime activity levels	0.64	0.43	0.02	1.08	0.31	0.03	0.28	0.60	0.01
Daytime sleep	**21.30**	**<0.000**	**0.39**	0.19	0.66	0.01	2.62	0.12	0.07
Assumed sleep	**3.98**	**0.05**	**0.11**	**4.49**	**0.04**	**0.12**	2.06	0.16	0.06
Actual sleep time	3.80	0.06	0.10	2.32	0.14	0.07	0.68	0.42	0.02
Sleep efficiency	**1.27**	**0.003**	**0.24**	1.14	0.29	0.03	2.35	0.13	0.07
Sleep latency	**1.25**	**0.003**	**0.24**	0.41	0.52	0.01	0.88	0.35	0.03
Immobile minutes	**4.65**	**0.04**	**0.12**	2.78	0.11	0.08	0.86	0.36	0.03
Fragmentation index	2.44	0.13	0.07	**4.93**	**0.03**	**0.13**	**4.67**	**0.04**	**0.12**

Statistical significances are shown in bold.

#### Daytime Activity Levels

Two-way ANOVA disclosed no main effects or interactions in daytime activity levels (Table 2). Correlation analysis showed a significant decrease in the fragmentation index with increasing daytime activity (*p* = 0.05, *r*^2^ = −0.1). Despite the significant correlation between the parameters, Pearson’s coefficient was weak.

#### Daytime Sleep

Two-way ANOVA disclosed significant shift typology effects with longer daytime sleep in the NS than in the DS group (*p* < 0.001, *d* = 1.4, *very large*; Tables 2, 3). Daytime sleep was inversely and significantly correlated with sleep efficiency and daytime activity levels (*p* = 0.03, *r*^2^ = −0.13; *p* = 0.05, *r*^2^ = −0.11, respectively). Longer daytime sleep was correlated with lower sleep efficiency and lower daytime activity levels. Despite the significant correlation between the parameters, Pearson’s coefficient was weak.

#### Sleep Parameters

Two-way ANOVA disclosed significant shift typology and chronotype effects for assumed sleep (Table 2). There was a statistically significant lower assumed sleep for the NS group (*p* = 0.05, *d* = 0.7, *medium*; Table 3); assumed sleep was shorter for the M-types than the N-types (M-type = 6.7 ± 0.7 vs. N-type = 7.2 ± 1.02, *p* = 0.04, *d* = 0.05, *medium*). Shift typology effects were significant for sleep efficiency, sleep latency, and immobile minutes (Table 2). A lower percentage of sleep efficiency (*p* = 0.003, *d* = 1.1, *very large*), longer sleep latency (*p* = 0.003, *d* = 1.1, *very large*), and more immobile minutes (*p* = 0.003, *d* = 0.8, *large*) were noted for the NS group compared to the DS group (Table 3).

Finally, two-way ANOVA uncovered chronotype effects and shift × chronotype interaction in the fragmentation index (Table 2). As regards the chronotype effect, a more fragmented sleep was noted for the N-types than the M-types (M-types = 24.1 ± 5.2 vs. N-types = 31.3 ± 4.4, *p* = 0.03, *d* = 1.5, *very large*). The *post hoc* tests for the interaction revealed a statistically significant higher fragmentation index for the NS M-types compared to the DS M-types (*p* = 0.02, *d* = 1.8, *very large*) and the DS N-types compared to the DS M-types (*p* = 0.01, *d* = 3.9, *very large*; Table 3).

**Figure 2:**
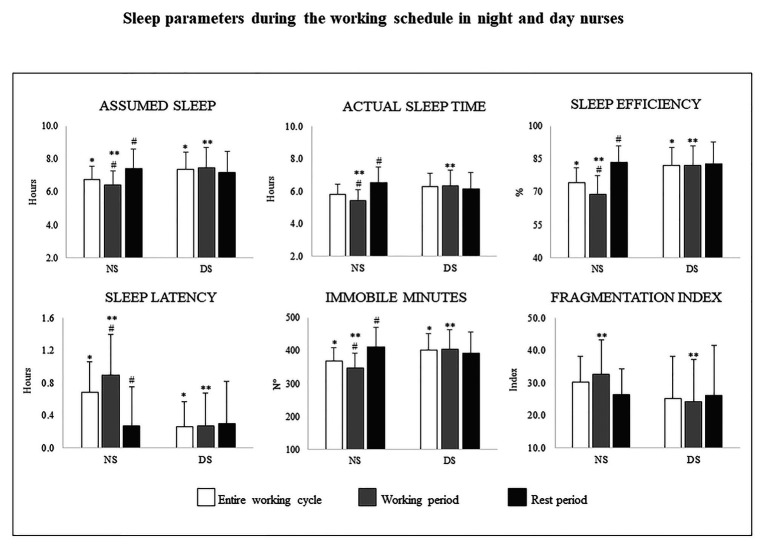
Mean ± SD for the assumed sleep and actual sleep time (decimal format), sleep efficiency (percentages), sleep latency (decimal format), immobile minutes (number), and fragmentation index during the entire working cycle, the working and the rest periods for the night shift (NS, on the left of each panel), and the day shift groups (DS, on the right of each panel). ^*^significant difference between NS and DS during the entire working cycle. ^**^significant difference between NS and DS during the Working period. ^#^significant difference between the working and the rest period for the NS group

In brief, the NS group spent less time sleeping, needed more time to fall asleep, and their overall sleep quality was lower compared to the DS group (Figure 2). In addition, sleep duration for the NS group was less than 7 h, and sleep efficiency was 10 percentage points below the 85% threshold (Hirshkowitz et al., 2015; Ohayon et al., 2017).

### Daytime Activity Levels, Daytime Sleep, and Sleep Parameters During the Working Period

Tables 4, 5 present the data for the Working Period.

**Table 4:** Actigraphy data (mean ± SD) recorded during the Working Period.

WORKING PERIOD						
	NS group			DS group		
Sleep parameters	Total (*n* = 24)	M-types (**n** = 9)	N-types (*n* = 15)	Total (*n* = 13)	M-types (*n* = 6)	N-types (*n* = 7)
Daytime activity levels (a.c.)	150.6 ± 35.76^*^	153.44 ± 43.49	147.75 ± 31.67	176.48 ± 35.52^*^	183.76 ± 45.53	169.18 ± 26
Daytime sleep (hours)	4.33 ± 1.58^*^	5.02 ± 1.75	3.93 ± 1.40	2.18 ± 1.83^*^	1.68 ± 1.28	2.63 ± 2.20
Assumed sleep (hours)	6.41 ± 0.85^*^	6.38 ± 0.53	6.43 ± 1.02	7.45 ± 1.25^*^	6.78 ± 0.52	8.02 ± 1.43
Actual sleep time (hours)	5.44 ± 0.67^*^	5.40 ± 0.55	5.45 ± 0.45	6.35 ± 0.95^*^	5.95 ± 0.33	6.70 ± 1.20
Sleep efficiency (%)	68.85 ± 8.57^*^	69.14 ± 8.87	68.68 ± 8.87	82.04 ± 9^*^	84.4 ± 4.03	79.67 ± 11.8
Sleep latency (hours)	0.90 ± 0.52^*^	0.82 ± 0.52	0.95 ± 0.53	0.27 ± 0.43^*^	0.18 ± 0.23	0.35 ± 0.57
Immobile minutes (n°)	346.56 ± 45.13^*^	343.96 ± 35.47	348.12 ± 51.19	404.34 ± 59.15^*^	379.66 ± 22.34	429.02 ± 72.56
Fragmentation index	32.65 ± .47^*^	32.73 ± 9.17^a^	32.14 ± 11.47	24.25 ± 13^*^	16.62 ± 8.54^a^^,^^b^	31.88 ± 12.47^b^

Daytime activity levels measured in activity counts (a.c.); daytime sleep and sleep parameters are reported in decimal format. Daytime sleep is calculated over the whole period (3 days); the other parameters refer to the single day. Results are presented separately for nurses working the night shift (NS) and those working only during the day (DS). The two groups are stratified by chronotype: Morning-types (M-types) and Neither-types (N-types).

* Two-way ANOVA – Shift typology effect.

a significant *post hoc* test for Two-way ANOVA– Shift typology × Chronotype interaction.

b significant *post hoc* test for Two-way ANOVA– Shift typology × Chronotype interaction.

**Table 5:** Two-way ANOVA analysis results with the two main effects (Shift typology and Chronotype) and the interaction (Shift typology × Chronotype) for the Working Period.

WORKING PERIOD – Two-way ANOVA									
Variable	Shift typology effect			Chronotype effect			Shift typology × Chronotype Interaction		
	*F*	*p*	*η_p_^2^*	*F*	*p*	*η_p_^2^*	*F*	*p*	*η_p_^2^*
Daytime activity levels	**4.17**	**0.05**	**0.11**	0.64	0.43	0.02	0.12	0.73	<0.000
Daytime sleep	**15.58**	**<0.000**	**0.33**	0.01	0.90	0.00	2.97	0.09	0.09
Assumed sleep	**8.51**	**0.006**	**0.21**	3.6	0.07	0.98	3.02	0.09	0.84
Actual sleep time	**10.91**	**0.002**	**0.25**	2.16	0.15	0.06	1.73	0.2	0.05
Sleep efficiency	**17.96**	**<0.000**	**0.35**	0.7	0.40	0.02	0.46	0.5	0.01
Sleep latency	**12.28**	**0.001**	**0.27**	0.73	0.40	0.02	0.01	0.96	<0.000
Immobile minutes	**11.4**	**0.002**	**0.26**	2.4	0.13	0.07	1.71	0.20	0.05
Fragmentation index	**4.77**	**0.04**	**0.13**	3.83	0.06	0.11	**4.48**	**0.04**	**0.12**

Statistical significances are shown in bold.

#### Daytime Activity Levels

**Figure 3:**
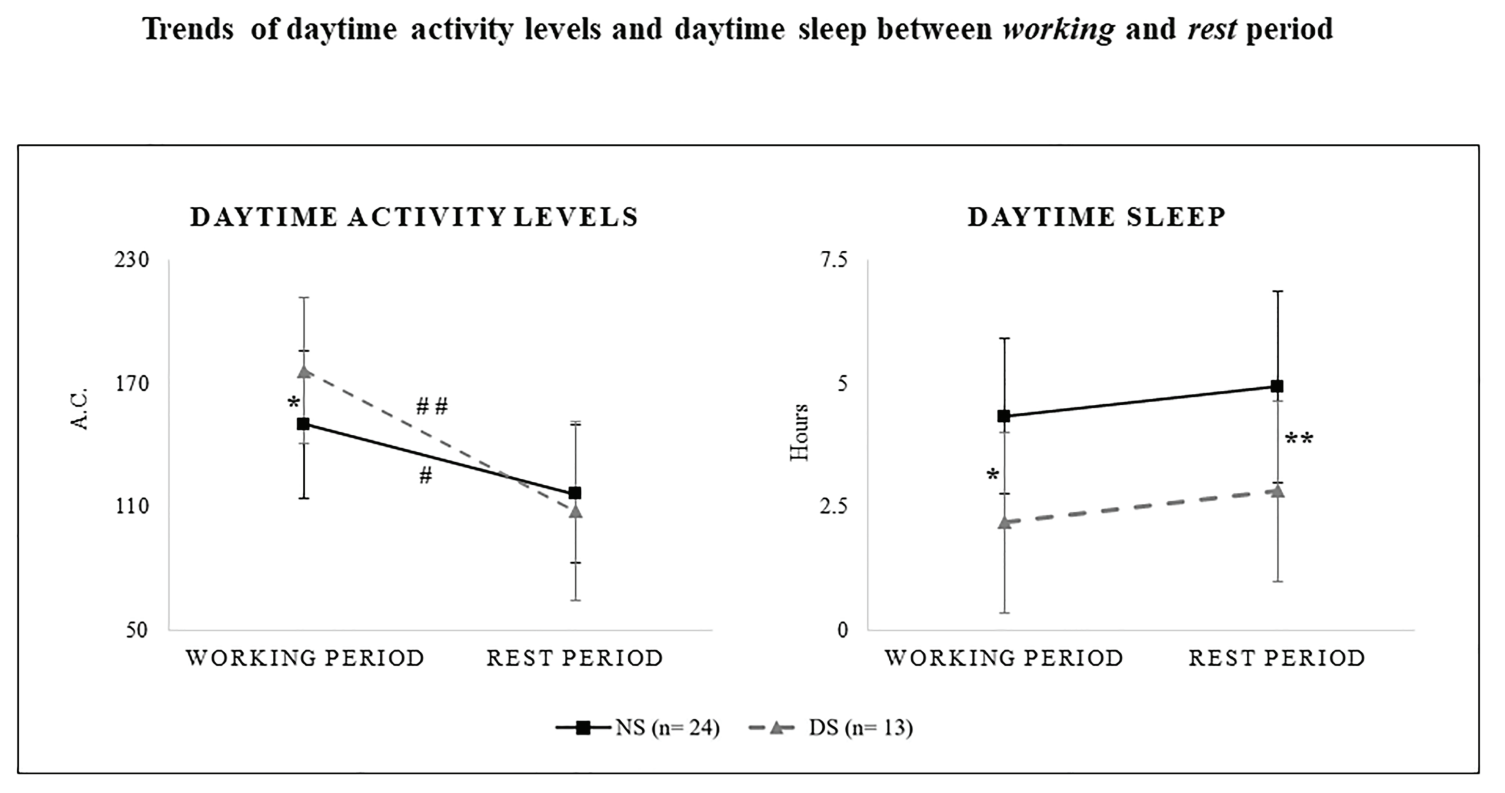
Daytime activity levels as measured in activity counts (a.c.; right panel) and daytime sleep (decimal format; left panel) during the working and the rest periods for the night shift (NS, solid line) the day shift group (DS, dashed line). ^*^significant difference between NS and DS during the working period. ^**^significant difference between NS and DS during the rest period. ^#^significant difference between the working and the rest period for the NS group.^##^significant difference between the working and the rest period for the DS group.

Two-way ANOVA disclosed a significant shift typology main effect (Table 5). Daytime activity levels were significantly lower in the NS than in the DS group (*p* = 0.05, *d* = 0.7, *medium*; Table 4; Figure 3).

#### Daytime Sleep

Two-way ANOVA revealed a significant shift typology effect for daytime sleep that was significantly longer for the NS than for the DS group (*p* = 0.03, *d* = 1.3, *very large*; Tables 4, 5; Figure 3). Daytime sleep was inversely correlated with average daytime activity for the NS group (*p* = 0.02, *r*^2^ = −0.14), indicating that longer daytime sleep reduced daytime activity levels. Despite the significant correlation results, Pearson’s coefficient was weak. Sleep efficiency was significantly decreased with longer daytime sleep for the DS group (*p* = 0.004, *r*^2^ = −0.55).

#### Sleep Parameters

Two-way ANOVA revealed a significant shift typology main effect for assumed sleep (*p* = 0.006, *d* = 1, *very large*), actual sleep time (*p* = 0.002, *d* = 1.2, *very large*), sleep efficiency (*p* < 0.000, *d* = 1.5, *very large*), sleep latency (*p* = 0.001, *d* = 1.3, *very large*), and the immobile minutes (*p* = 0.002, *d* = 1.1, *very large*; Table 5). The NS group slept less, reported lower sleep efficiency, took more time to fall asleep, and recorded fewer immobile minutes than the DS group (Table 4; Figure 2).

Finally, two-way ANOVA uncovered shift typology effects and shift × chronotype interaction in the fragmentation index (Table 5). Regarding the shift typology effect, fragmented sleep was greater for the NS group than the DS group (*p* = 0.04, *d* = 0.7, *medium*). The *post hoc* tests for the interaction revealed a statistically significant higher fragmentation index for the NS M-types compared to the DS M-types (*p* = 0.008, *d* = 4.8, *very large*) and for the DS N-types compared to the DS M-types (*p* = 0.01, *d* = 3.6, *very large*), and (Table 4; Figure 2).

### Daytime Activity Levels, Daytime Sleep, and Sleep Parameters During the Rest Period

Tables 6, 7 present the data recorded during the Rest Period.

**Table 6:** Actigraphy data (mean ± SD) recorded during the Rest Period.

REST PERIOD						
	NS group			DS group		
Sleep parameters	Total (*n* = 24)	M-types (*n* = 9)	N-types (*n* = 15)	Total (*n* = 13)	M-types (*n* = 6)	N-types (*n* = 7)
Daytime activity levels (a.c.)	116.92 ± 33.77	120.4 ± 43.43	113.44 ± 27.95	108.49 ± 43.51	120.55 ± 56.52	96.44 ± 28.47
Daytime sleep (hours)	4.93 ± 1.93^*^	5.65 ± 1.9	4.47 ± 1.87	2.82 ± 1.82^*^	2.23 ± 1.9	3.32 ± 1.72
Assumed sleep (hours)	7.43 ± 1.15	7 ± 1.25	7.70 ± 1.05	7.18 ± 1.28	6.9 ± 0.97	7.43 ± 1.55
Actual sleep time (hours)	6.53 ± 0.97	6.17 ± 1.02	6.75 ± 0.88	6.13 ± 1.03	6.13 ± 0.80	6.15 ± 1.27
Sleep efficiency (%)	83.56 ± 7.49	80.58 ± 8.95	86.54 ± 5.63^a^	82.07 ± 9.97	87.35 ± 4^b^	76.78 ± 11.11^a^^,^ ^b^
Sleep latency (hours)	0.27 ± 0.48	0.53 ± 0.73^a^	0.10 ± 0.08^a^	0.3 ± 0.52	0.08 ± 0.13	0.48 ± 0.65
Immobile minutes (n°)	411.4 ± 60.4	388.3 ± 67.4	425.3 ± 53.3	391.7 ± 65	386.8 ± 53.9	396 ± 77.3
Fragmentation index	26.3 ± 8.1	26.2 ± 6.7	26.3 ± 9.1	26 ± 15.6	19.6 ± 8.2	31.4 ± 18.8

Daytime activity levels measured in activity counts (a.c.); daytime sleep and sleep parameters are reported in decimal format. Daytime sleep is calculated over the whole period (2 days); the other parameters refer to the single day. Results are presented separately for nurses working the night shift (NS) and those working only during the day (DS). The two groups are stratified by chronotype: Morning-types (M-types) and Neither-types (N-types).

* Two-way ANOVA – Shift typology effect.

a significant *post hoc* test for Two-way ANOVA– Shift typology × Chronotype interaction.

b significant *post hoc* test for Two-way ANOVA– Shift typology × Chronotype interaction.

**Table 7:** Two-way ANOVA analysis results with the two main effects (Shift typology and Chronotype) and the interaction (Shift typology × Chronotype) for the Rest Period.

REST PERIOD – Two-way ANOVA									
Variable	Shift typology effect			Chronotype effect			Shift typology × Chronotype Interaction		
	*F*	*p*	*η_p_^2^*	*F*	*p*	*η_p_^2^*	*F*	*p*	*η_p_^2^*
Daytime activity levels	0.41	0.53	0.01	1.40	0.25	0.04	0.43	0.52	0.01
Daytime sleep	**12.27**	**<0.000**	**0.28**	0.01	0.93	0.00	3.05	0.09	0.09
Assumed sleep	0.19	0.67	0.01	2.16	0.15	0.06	0.05	0.83	0.00
Actual sleep time	0.83	0.37	0.02	0.77	0.39	0.02	0.65	0.43	0.02
Sleep efficiency	0.32	0.58	0.01	0.76	0.39	0.02	**9.80**	**<0.000**	**0.23**
Sleep latency	0.05	0.83	0.00	0.03	0.86	0.00	**6.52**	**0.02**	**0.17**
Immobile minutes	0.51	0.48	0.02	1.14	0.29	0.03	0.41	0.53	0.01
Fragmentation index	0.04	0.85	0.00	2.45	0.13	0.07	2.31	0.14	0.07

Statistical significances are shown in bold.

#### Daytime Activity Levels

Two-way ANOVA showed no statistically significant interactions or main effects (Table 7; Figure 3).

#### Daytime Sleep

Two-way ANOVA showed a statistically significant shift typology effect in daytime sleep, which was significantly longer for the NS group compared to the DS group (*p* = 0.03, *d* = 0.8, *large*; Tables 6, 7; Figure 3). Daytime sleep in the NS group was inversely correlated with daytime activity levels (*p* = 0.03, *r*^2^ = −0.2). Despite the significant correlation between the parameters, Pearson’s coefficient was weak.

#### Sleep Parameters

Two-way ANOVA showed a statistically significant interaction for sleep efficiency and sleep latency (Table 7). The *post hoc* tests showed higher values of sleep efficiency for the NS N-types compared to the DS N-types (*p* = 0.01, *d* = 1.3, *very large*) and for the DS M-types compared to the DS N-types (*p* = 0.02, *d* = 1.2, *very large*). Sleep latency was shorter for the NS M-types compared to the NS N-types (*p* = 0.03, *d* = 1, *very large*; Table 6). In general, the NS group slept slightly longer (see actual sleep time) and better (see sleep efficiency and immobile minutes) than the DS group, and sleep efficiency was still below 85% when considering the entire sample (Figure 2).

### Comparison Between Working and Rest Period in Daytime Activity Levels, Daytime Sleep, and Sleep Parameters

#### Daytime Activity Levels

Three-way ANOVA shift typology × periods × chronotype of daytime activity levels showed a statistically significant shift typology × periods effect (*F* = 10.7, *p* < 0.001, *η_p_*^2^ = 0.33). The *post hoc* tests were significant, showing lower levels during the Rest Period compared to the Working Period for both the NS and the DS group (*p* = 0.02, *d* = −1, *very large*; *p* < 0.001, *d* = −1.2, *very large*). The difference between the Working and the Rest period was a decrease of 33.7 activity counts for the NS group and of 67.9 activity counts for the DS group (Figure 3).

#### Daytime Sleep

Three-way ANOVA shift typology × periods × chronotype of sleep recovery showed no statistically significant interaction (*F* = 0.02, *p* < 0.89, *η_p_*^2^ < 0.001).

#### Sleep Parameters

Three-way ANOVA shift typology × periods × chronotype revealed a significant interaction between shift typology × periods for assumed sleep (*F* = 4.72, *p* = 0.03, *η_p_*^2^ = 0.7), actual sleep time (*F* = 7.55, *p* = 0.008, *η_p_*^2^ = 0.10), sleep efficiency (*F* = 12.91, *p* = 0.001, *η_p_*^2^ = 0.16), sleep latency (*F* = 5.93, *p* = 0.02, *η_p_*^2^ = 0.08), and immobile minutes (*F* = 7.1, *p* = 0.01, *η_p_*^2^ = 0.09). The *post hoc* tests disclosed for the NS group statically significant less assumed sleep (*p* = 0.02, *d* = 0.9, *large*), actual sleep time (*p* < 0.000, *d* = 1.1, *very large*), sleep efficiency (*p* < 0.000, *d* = 4.6, *very large*), fewer immobile minutes (*p* = 0.001, *d* = 1, *very large*), and longer sleep latency (*p* < 0.000, *d* = 1.3, *very large*) during the working period compared to the rest period (Figure 2). There was a significant chronotype main effect for assumed sleep and fragmentation index: assumed sleep was longer for the N-types compared to the M-types (M-types = 7.2 ± 1 vs. N-types = 7.85 ± 1.2, *F* = 5.45, *p* = 0.02, *η_p_*^2^ = 0.8, *d* = 3.5, *very large*); sleep was less fragmented for the M-types compared to the N-types (M-types = 23.77 ± 2.02 vs. N-types = 30.44 ± 1.75, *F* = 6.19, *p* = 0.01, *η_p_*^2^ = 0.09, *d* = 3.8, *large*).

## Discussion

With the present study, we wanted to objectively assess daytime activity, daytime sleep, and sleep parameters in hospital staff nurses. To our knowledge, this is one of the first studies to link daytime sleep with daytime activity in shift workers. The main findings are that during the Working Period, the daytime activity levels were lower for the NS compared to the DS group, while they were similar during the Rest Period. The time spent resting in daytime sleep was considerably longer for the NS compared to the DS group during both the Working and the Rest periods. It seems that both the NS and the DS group tended to accumulate an activity debt during their working schedule. With the term activity debt, we refer to the decrease in daytime activity level between the Working and the Rest period, and the inability to maintain a constant level of activity between the two. This reduction, accumulated over time during each work cycle, has negative effects on activity levels. The activity debt could stem from the difficulty of maintaining a daytime activity level during the Rest Period similar to that of the Working Period.

Consequently, the activity debt was greater in the NS group because their daytime activity levels were lower compared to the DS group also during the Working Period. The activity debt might also be linked to the longer daytime sleep, the NS group needed to reduce their night shift sleep debt. So, while daytime sleep could help the NS group make up for the loss of sleep, it resulted in less activity. During the Entire Working Cycle and the Working Period, sleep parameters were generally worse in the NS compared to the DS group probably due to the night shift.

### Daytime Activity Levels and Daytime Sleep

The daytime activity levels of the NS group were lower during the Rest Period compared to the Working Period and daytime sleep was longer during the Working compared to the Rest Period. The extended rest the NS group took may have been longer during the Rest Period because the nurses had more free time to rest. We may speculate that the NS group took advantage of their daytime sleep to reduce the sleep debt accumulated from working night shifts.

A similar trend was noted for the DS group: daytime activity levels were higher during the Working Period, while daytime sleep was longer during the Rest Period. We may assume that, because of the workload while on duty or the early wake-up time when on the morning shift, the DS group accumulated physical fatigue and sleepness that probably needed to be compensated by daytime sleep during the work-off days. Taken together, the Rest Period (when physical effort is most likely less than during the Working Period) and the longer daytime sleep duration reduced the daytime activity levels, resulting in higher values for the DS group than the NS group. Hence, also the DS group needed daytime sleep to compensate for the workload effort.

The daytime activity levels during Working Period were higher for the DS than the NS group probably because the DS group had less time for daytime sleep during the day. The NS group rested about 2 h more than the DS group. This longer daytime sleep reduced their daytime activity levels, as shown in the correlation analysis. In essence, it appears that the more time the nurses spent in daytime sleep, the lower their daytime activity levels.

Night shift workers are noted to recover by sleeping longer and more often (Akerstedt, 2003; Karhula et al., 2013; Korsiak et al., 2018; Reid et al., 2018). Reid et al. (2018) reported that night and irregular shift workers had longer sleep recovery during the day compared to day workers; they concluded that sleeping during the day might be a way to compensate for chronic sleep loss (Reid et al., 2018). In line with Korsiak et al. (2018) and Reid et al. (2018) reported that sleeping during the afternoon could compensate for the differences in sleep duration between day and night workers. This may mean that sleep recovery during the daytime results from shortened or disturbed nighttime sleep (Korsiak et al., 2018). Consistent with this previous study, our data showed an increase in daytime sleep time when sleep efficiency was low. This may indicate that daytime sleep is needed when sleep quality is insufficient. At any rate, longer daytime sleep resulted in lower daytime activity levels.

During the Rest Period, daytime activity levels for the NS group were significantly decreased, while daytime sleep was about 30 min longer than during the Working Period. Similarly, the DS group’s daytime activity levels were significantly lower compared to the levels recorded during the Working Period. However, the NS group was less active probably because it spent more time sleeping to compensate for the sleep debt during the afternoon before the night shift. What was unexpected was the drop in daytime activity levels between the Working and Rest period for the DS group.

This last point indicated that the shift working schedule could affect the DS group’s daytime activity levels, even though they did not work the night shift. The DS group probably also experienced sleep problems during the working cycle and needed to recover during the Rest Period. One possible explanation could be that the very early wake-up call to arrive at work on time for the morning shift forces nurses to stay awake longer than when they work the afternoon shift. While the DS group spent less time in daytime sleep, its daytime activity level was comparable to that of the NS group. Accordingly, we may speculate that the workload led to a need for recovery like that of the NS group.

As the present study is one of the first to measure daytime activity levels by actigraphic monitoring, it is difficult to say whether the daytime activity levels in our sample are high, low, or merely average. In their study, Chang and Li (2019) reported daytime activity levels calculated over 5 workdays (excluding rest days) of nurses working the same work shift for a month (Chang and Li, 2019). Due to the difference in the way the work period was split, a comparison between our results and the daytime activity levels reported by Chang and Li (2019) might be misleading. Nonetheless, the daytime activity levels for the DS group during the working period were similar to those reported by Chang and Li, who found no difference in daytime activity levels (Chang and Li, 2019). This observation is in line with our results for the entire working cycle. Nonetheless, the different subdivision of the working periods precludes comparison between our study and theirs.

Daytime sleep and daytime activity levels were inversely weakly correlated only for the NS group. As mentioned above, a plausible explanation is that longer daytime sleep is needed to reduce sleep debt, particularly during off-duty days. In their study involving Brazilian nurses, Ribeiro-Silva et al. (2006) reported that workplace napping helped keep the total sleep duration similar to the other nights when sleep and nap length were added together.

Overall, during the entire working cycle, the NS group spent approximately 12 h in recovery strategies, about 2.1 h of sleep during the night-shift and about 9.33 h of daytime sleep. We can assume that it is more a matter of sleep duration than sleep quality of almost average values. Ruggiero and Redeker (2014) and Davy and Göbel (2018) also advanced this hypothesis in their studies on shift workers.

### Sleep Parameters

Analysis of the data for the entire working cycle and working period showed low sleep parameters for the NS group. The sleep parameters were below the good sleep threshold, with sleep efficiency <85% (Ohayon et al., 2017) and sleep duration <7 h per night (Hirshkowitz et al., 2015). When working the night shift, the sleep parameters were worse for the NS group compared to the DS group. Reduced sleep quality in night shift workers has been variously investigated (Ribeiro-Silva et al., 2006; Kecklund and Axelsson, 2016; van de Ven et al., 2016; Korsiak et al., 2018; Reid et al., 2018; Chang and Li, 2019; Uekata et al., 2019). Our data for the entire working cycle show, however, that sleep fragmentation was not necessarily greater for the NS than the DS group.

Of note is that the analysis of the Working Period included the afternoon shift. Sleep duration is usually longer on the nights before and after the afternoon shift due to the later work shift start time and the possibility to sleep longer in the morning (Akerstedt, 2003; Karhula et al., 2013; Baek et al., 2020).

A considerable increase in most of the sleep parameters between the Rest and the Working period was observed for the NS group: sleep duration was longer, sleep efficiency greater, immobile minutes longer, and sleep latency shorter. The improvement in overall sleep quality was probably due to the absence of night shift work. What should be taken into account is that the last day of the rest period involves an early wake-up-time for the following morning shift (Akerstedt, 2003; Sallinen and Kecklund, 2010). As Akerstedt (2003) explained, the morning shift requires a very early wake-up in the morning and forces nurses to stay awake longer than usual (Akerstedt, 2003). Morning shift entails non-spontaneous awakening around 4:00–5:00. This, coupled with the inability to go to bed earlier, reduces sleep duration by approximately 2–4 h and forces shift workers to stay awake 2–3 h longer than usual, which increases sleepiness and raises the risk of accidents and work errors that can compromise patient care (Surani et al., 2015; Li et al., 2019).

Not working during the night, the DS group showed fewer or no variations in sleep parameters. But they did not always sleep 7–8 h, sleep efficiency remained below the 85% threshold, and the amount of immobile minutes was significantly lower compared to the NS group. During the working period, the sleep parameters were significantly worse for the NS than the DS group, whereas the sleep quality was similar for both groups with no remarkable differences in sleep parameters during the rest period. When off-work, both groups were freer to set their personal sleep times.

### Role of Chronotype

In our sample, E-types were absent (the only subject classified as E-type during recruitment was excluded from the analysis). There are two possible explanations for this: first, the sample size was too small to identify E-types, and second, the change in preference towards N-or M-type as the chronotypes adjusted their circadian rhythm to irregular work schedules. This made it impossible to compare the two chronotypes. In a previous study, Korsiak et al. (2018) also underlined the lack of differences between M- and N-types working the same shift. Although their sample was larger than ours and included different chronotypes, the differences were visible only for the E-types (Korsiak et al., 2018).

In general, the differences between chronotypes were similar to those of the two groups considered as a whole. While it may seem that the N-types slept longer, M-type sleep is sometimes more efficient and less fragmented. Though not supported by statistical significance in every instance, we may hypothesize that the M-types in the NS group probably endured more stress from night work than the M-types in the DS group, whereas the N-types in the DS group were at a disadvantage because they were less active and slept longer the daytime. The data from a larger sample could help to strengthen our findings.

Previous studies support our hypothesis: morningness does not set well with night duty and night work probably due to the asynchrony between circadian rhythms of morning chronotypes and the activation timing of night work (van de Ven et al., 2016; Zion et al., 2018; Hittle and Gillespie, 2019). The lack of significance for NS chronotypes could be explained by the small sample size in the present study.

### Study Limitations

This study has several limitations. First, we recorded only one work cycle. While we are aware that optimal duration of monitoring should have included at least two work cycles, the shortened monitoring was practical trade-off to ensure compliance and effective monitoring, and minimize the risk of potential dropout. Second, though the sample was small, the statistical power was enough to guarantee statistical significance. The small sample size could have resulted in under-representation of the three chronotypes (not allowing appropriate comparison between M- and E-types) and the correlation analysis weakness. Studies with a larger sample size are needed to strengthen the correlations between daytime sleep and activity levels. The assumption of a relationship between daytime sleep and daytime activity levels should be treated with caution pending future studies to support these conclusions. Furthermore, as concerns chronotype assessment, the absence of E-types could be addressed by use of the MEQ instead of a more specific questionnaire, such as the Munich Chronotype Questionnaire for shift workers (Juda et al., 2013).

Finally, women are overrepresented in our sample from a single hospital, reflecting the gender composition of the nursing workforce in Italian hospitals. We did not collect information about marital status and family composition, which could have added details about non-work commitments of the nurse in this sample.

## Conclusion and Perspectives

Based on daytime sleep and daytime activity levels, we may conclude that sleeping during the daytime may be a good strategy for night-shift workers to counteract the sleep debt accumulated during night work and for day nurses to recover from the daily workload. This can reduce daytime activity levels, however, since the more the rest taken during the daytime, the lower the daytime activity levels. Day by day, night shift workers accumulate an activity debt due to less daytime activity compared to day nurses, particularly during working days. Besides desynchronization of circadian rhythm, reduced daytime activity could seriously compromise shift worker health (Atkinson et al., 2008): a less fit or active person is more at risk of developing chronic disease, cardiovascular diseases, and metabolic problems (Warburton, 2006; Blair et al., 2012). A future area of focus is to improve daily activity levels and provide nurses with practical suggestions for better sleep hygiene to improve their quality of life.

## Data Availability Statement

The raw data supporting the conclusions of this article will be made available by the authors, without undue reservation, to any qualified researcher.

## Ethics Statement

The studies involving human participants were reviewed and approved by Ethical Committee of the San Raffaele Hospital (CE: 156/int./2017), and registered at ClinicalTrials.gov (registration number: NCT03453398). The patients/participants provided their written informed consent to participate in this study.

## Author Contributions

ER: conceptualization, methodology, resources, data curation, writing – original draft, writing – review and editing, and supervision. LC: investigation, formal analysis, data curation, writing – original draft, writing – review and editing, and visualization. LG: methodology, investigation, formal analysis, data curation, writing – original draft, writing – review and editing, and visualization. AMu: investigation, resources, writing – original draft, writing – review and editing, and visualization. EC: conceptualization, methodology, investigation, writing – original draft, writing – review and editing, supervision, and visualization. VC: formal analysis, writing – original draft, and writing – review and editing. GB and FE: conceptualization, methodology, writing – original draft, writing – review and editing, and supervision. AMo: conceptualization, methodology, data curation, writing – original draft, and writing – review and editing. All authors contributed to the article and approved the submitted version.

### Conflict of Interest

The authors declare that the research was conducted in the absence of any commercial or financial relationships that could be construed as a potential conflict of interest.

## Abbreviations

DS: Diurnal shift
NS: Night shift
M-type: Morning-type
N-type: Neither-type
E-type: Evening-type
